# Non-periodic outbreaks of recurrent epidemics and its network modelling

**DOI:** 10.1038/srep16010

**Published:** 2015-11-02

**Authors:** Muhua Zheng, Chaoqing Wang, Jie Zhou, Ming Zhao, Shuguang Guan, Yong Zou, Zonghua Liu

**Affiliations:** 1 Department of Physics, East China Normal University, Shanghai, 200062, China; 2 College of Physics and Technology, Guangxi Normal University, Guilin 541004, China

## Abstract

The study of recurrent epidemic outbreaks has been attracting great attention for decades, but its underlying mechanism is still under debate. Based on a large number of real data from different cities, we find that besides the seasonal periodic outbreaks of influenza, there are also non-periodic outbreaks, i.e. non-seasonal or non-annual behaviors. To understand how the non-periodicity shows up, we present a network model of SIRS epidemic with both time-dependent infection rate and a small possibility of persistent epidemic seeds, representing the influences from the larger annual variation of environment and the infection generated spontaneously in nature, respectively. Our numerical simulations reveal that the model can reproduce the non-periodic outbreaks of recurrent epidemics with the main features of real influenza data. Further, we find that the recurrent outbreaks of epidemic depend not only on the infection rate but also on the density of susceptible agents, indicating that they are both the necessary conditions for the recurrent epidemic patterns with non-periodicity. A theoretical analysis based on Markov dynamics is presented to explain the numerical results. This finding may be of significance to the control of recurrent epidemics.

Epidemic spreading has been a challenging problem for a long time and become very hot again in the recent decade, mainly because of the fast growing of network science. It is revealed that the network structure plays a key role in this process, especially in the aspect of epidemic threshold^1^,^2^,^3^,^4^,^5^,^6^,^7^,^8^. So far, the studying of epidemic in complex networks has undergone three stages. In the first stage, the attention was focused on the static networks where each node represents an immobile agent and the contagion occurs only between the neighboring nodes through links. It was interestingly revealed that for scale-free networks, the epidemic threshold will be vanishingly small in the thermodynamic limit^1^,^2^. In the second stage, the attention was moved to the reaction-diffusion model where agents can move to their neighboring nodes with a possibility^9^,^10^,^11^,^12^. In this framework, the contagious process takes place only within the agents at the same node and the links are used only for diffusion. In the third stage, the research interest concentrated on how the concrete factors influence the epidemic spreading, such as the objective traveling of human being^13^,^14^,^15^, the interplay between epidemic spreading and network structure^16^,^17^, the traffic-driven epidemic spreading^18^,^19^,^20^,^21^, and the case of multilayer networks and temporal networks^22^,^23^,^24^,^25^,^26^,^27^,^28^,^29^,^30^,^31^,^32^,^33^,^34^ etc. These models significantly increase our understanding on epidemic spreading and are very useful for public health authorities to assess situations quickly, make informed decisions, and optimize vaccination and drug delivery plans etc.

All the above studies are focused on the case of a single outbreak of epidemic and its dependence on parameters such as the network topology, received information and diffusion mode etc. However, there is another parallel line on recurrent outbreaks of epidemic although it is not hot so far. Its study is undoubtedly significant for preventing the recurrence of the new emergent viruses such as SARS (Severe Acute Respiratory Syndrome), H1N1 (Swine Influenza), H5H1 (Avian Influenza), Ebola, and MERS (Middle East Respiratory Syndrome) etc. The previous studies in this line are mainly focused on the recurrence of seasonal influenza which recurs annually in most temperate climatic zones of the world^35^,^36^,^37^,^38^,^39^,^40^,^41^,^42^. Typically, serious epidemics occur in winter or spring followed by fade-out periods during the warmer months. The seasonal influenza is also a source of considerable human mortality, reaching some 250,000 to 500,000 deaths per year globally. Even today in the United States, a widespread flu over 46 states caused 26 children dead in January, 2015^43^. To understand the mechanism of the recurrence of seasonal influenza, Ref. 35 examined the spatial-temporal dynamics of DHF (Dengue Hemorrhagic Fever) incidence in a data set describing 850,000 infections occurring in 72 provinces of Thailand during the period 1983 to 1997. Ref. 36 simulated the recurrence of epidemic by considering the infection rate 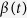 as a sinusoidal function. Refs 38,40 analyzed the daily influenza-like illness cases reported in Israel. Ref. 39 studied the spatio-temporal patterns of influenza owing to the presence of nonstationarity and nonlinearity in incidence data. Although these progresses are significant, there is still controversy in identifying the seasonal drivers that generate annual influenza oscillations. Especially, little attention has been paid to the influence of network topology.

To take a further step to identify the drivers of seasonal influenza and to study the influence of network topology on the recurrence of epidemic, we recently collect the influenza data from Hong Kong^44^. Very interestingly, we find that the outbreak of influenza is not always seasonal and there is even no outbreak in some years, in contrast to the annual outbreaks studied in the past^35^,^36^,^37^,^38^,^39^,^40^. We have also found this unexpected phenomenon in other cities such as in Baltimore and New York etc^45^, indicating that the irregular outbreak is generic. This irregularity hampers us to make long-term predictions of infectious diseases and thus motivates us to study its underlying mechanism. In this paper, we present a network model of SIRS epidemic with both time-dependent infection rate and a small possibility of persistent epidemic seeds, representing the influences from the larger annual variation of environment and the infection generated spontaneously in nature, respectively. Our numerical simulations reveal that the model can show the main features of real influenza data. Further, we find that the recurrent outbreak of epidemic depends not only on the infection rate but also on the density of susceptible agents, indicating that they are both the necessary conditions for the recurrent epidemic patterns with non-periodicity. A theoretical analysis based on Markov dynamics is presented to explain the numerical results. This finding may be of significance to the long-term prediction and control of recurrent epidemics.

## Results

### Non-periodicity of recurrent influenza data

**Figure 1:**
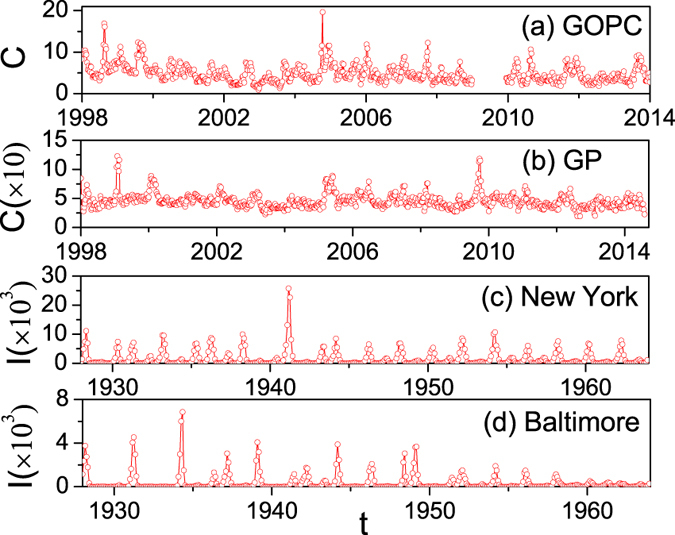
Time series of number of influenza viruses detected. (**a**,**b**) represent the weekly consultation rates of influenza-like illness (per 1000 consultations) in Hong Kong from the General Out-Patient Clinics (GOPC) and the General Practitioners (GP), respectively, where the data from 2009/6/13 to 2010/5/23 in (**a**) are not available. The value of *C* in (**b**) is from 0 to 150. (**c**,**d**) represent the time series of reported measles infective cases *I* in New York and Baltimore, respectively. The variable *I* in (**c**) is from 0 to 3×10^4^ and that in (**d**) is from 0 to 8×10^3^.

Figure 1(a,b) show the weekly consultation rates of influenza-like illness (per 1000 consultations) collected from the sentinel points involving General Practitioners (GP) (Fig. 1(a)) and General Out-Patient Clinics (GOPC) (Fig. 1(b)) under the sentinel surveillance system in Hong Kong^44^, where the data from 2009/6/13 to 2010/5/23 in (a) was not collected by the Centre. The value of *C* in (b) is from 0 to 150. These two sets of data are highly correlated, see Fig. 1 in SI. It means that the weekly consultation rates of influenza-like illness can well reflect the overall influenza-like illness activity. However, limited by the records, the data in Fig. 1(a,b) did not distinguish the types of influenza viruses. Fortunately, the Department of Health of Hong Kong made a classification of the influenza viruses in recent years. The Table-I in SI shows the components of influenza viruses in the years from 2010 to 2013, where the tested specimen obtained from GOPCs, GPs, public and private hospitals. From this Table we see that they are mainly concentrated on three kinds of typical viruses, indicating that the influenza data in Fig. 1(a,b) can represent the features of a typical influenza virus.

**Figure 2:**
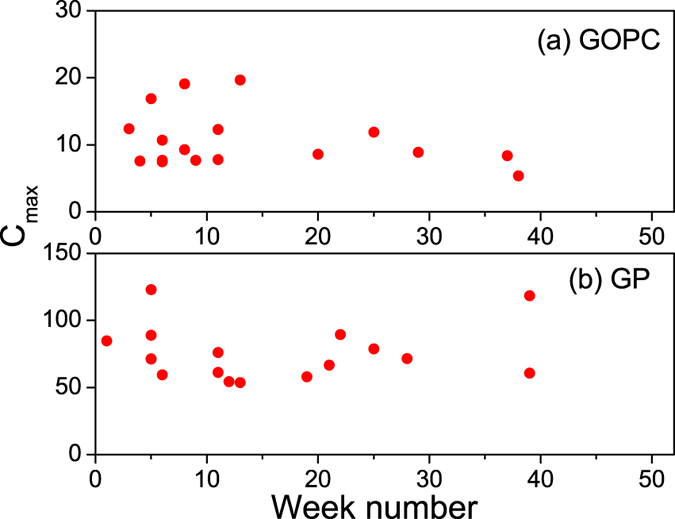
The maximum weekly consultation rates in each year corresponding to Fig. 1(a,b), respectively.

From Fig. 1(a,b) we interestingly find that the peak of the weekly consultation rates of influenza-like illness fails to appear in some years and the intervals between two consecutive peaks are not very regular, in contrast to the regularity of annual outbreaks reported in refs 35, 36, 37, 38, 39, 40, 41. To make it clear, we select the maximum weekly consultation rates in each year from Fig. 1(a,b) and plot them in Fig. 2(a,b), respectively. From Fig. 2(a,b) one can easily find that the points are not distributed only in the weeks of winter or spring but distributed in most of the 52 weeks of a year, indicating the feature of non-periodicity. Is this a specific phenomenon only in Hong Kong? To figure out the answer, we have checked a large number of other recurrent influenza data and found that such phenomenon also shows up in other cities. Figure 1(c,d) show two such examples of measles infective cases I in New York and Baltimore, respectively. It is easy to see that their outbreaks are also non-periodic, indicating that this non-periodicity is generic in recurrent influenza data. On the other hand, we find that the average of Fig. 1(a,b) over the whole 16 years is an oscillatory behavior but not an unimodal distribution, see Fig. 2 in SI, supporting the feature of non-periodicity again.

### A network model to reproduce the non-periodic epidemic patterns

Epidemic spreading is usually studied by the classic epidemic models^46^ such as the susceptible-infected-susceptible (SIS) model and the susceptible-infected-refractory (SIR) model. In an isolated SIS model, a susceptible node may be infected by an infected neighbor at rate *β*. In the meantime, each infected node will become susceptible again at rate *μ* at each time step. After the transient process, the system reaches a stationary state with a constant infected density *I*, i.e. having no decreasing process and thus no oscillatory behavior. Thus, the SIS model cannot be used to describe the recurrent epidemic patterns. Similarly, in an isolated SIR model, a susceptible node may be infected by an infected neighbor at rate *β*. At the same time, the infected node will decay into a refractory one with probability *μ* at each time step. The infection process will be over when there is no infected *I*, implying that the refractory density *R* monotonously increases but never drops down. Thus, the SIR model cannot be used to describe the recurrence of influenza data. To successfully reproduce the recurrent outbreaks, ref. 36 considered a specific SIR model with a time-dependent infected rate 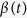, i.e. a sinusoidal function of time *t*, where both the birth rate and mortality rate are included. As the mortality rate will make the refractory *R* decrease and the birth rate will make the susceptible *S* increase, thus this SIR model is in fact equivalent to the susceptible-infected-refractory-susceptible (SIRS) model^47^. This work is significant in revealing the regular outbreak of epidemic, but it fails to explain the non-periodicity observed in Figs 1 and 2.

**Figure 3:**
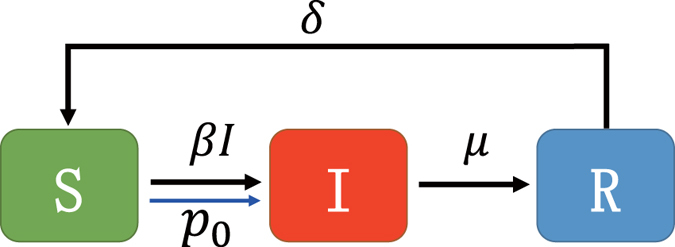
Schematic figure of our SIRS model. The symbols *S*, *I* and *R* represent the susceptible, infectious, and refractory states, respectively. The parameters *β*, *μ* and *δ* represent the infection, refractory and recovery rates, respectively. 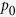 represents the probability for a susceptible person to be naturally infected by the environment and other factors. When *β* is small, the infection probability of a susceptible person is proportional to both the infected neighbors *I* and the infection rate *β*. Thus, the total probability for a susceptible person to be infected is approximately 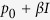. At the same time, an infectious person will have a probability *μ* to become refractory and a refractory person will have a probability *δ* to recover to the susceptible.

To understand the mechanism of the non-periodicity in the recurrent influenza data, we here propose a SIRS model to reproduce the epidemic patterns with non-periodicity. Figure 3 shows its schematic figure. In this model, we have included two characteristic features: one is the time-dependent infected rate 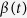 from one year to another, i.e. piecewise constants (see *Methods* for details), and the other is a small natural infection rate 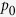. The former comes from the observation in Table-I in SI that the components of viruses are different from one year to another. It is well known that different viruses have different infection rates. If we use a single infection rate *β* to represent the comprehensive effect of all the components of viruses in one year, the value of *β* will be thus different from one year to another, indicating that *β* depends on time *t* and can be taken as different constants in different years. This is different from the sinusoidal function in ref. 36. The latter comes from the fact that there is always a small fraction of naturally infected people in our society, which may come from the environment. That is, there is a small probability 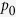 to generate infected seeds at each time step, in contrast to the previous models with only initial seeds.

**Figure 4:**
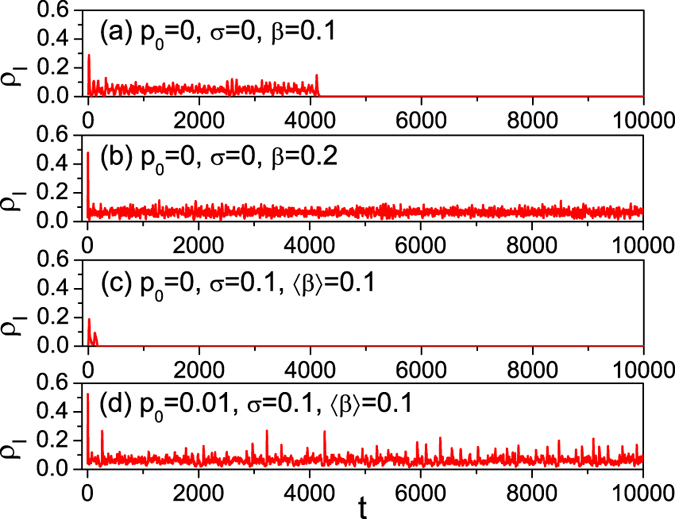
Evolution of infected density *ρ*_*I*_ for different sets of parameters. (**a**) Case of constant infection rate with 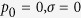, and 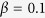; (**b**) Case of constant infection rate with 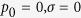, and 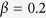; (**c**) Case of time-dependent infection rate with 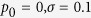, and 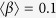; (**d**) Case of both time-dependent infection rate and nonzero 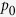 with 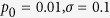, and 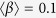.

We let the network be the uncorrelated configuration model (UCM) with a power-law degree distribution 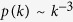, size 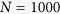, and average degree 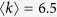
48, see *Methods* for details. The dynamics equation of SIRS model is also given in the section of *Methods*. Numerical simulations of this model show that the recurrent behaviors of non-periodic epidemic patterns can be reproduced only when we take a time-dependent 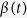 and a nonzero 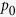. Let 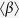 represent the average of 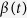 and *σ* be its standard deviation. Figure 4(a,b) shows the results of constant infection rate with 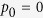 and 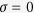 where we have 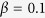 in (a) and 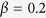 in (b). Figure 4(c,d) shows the results of time-dependent infection rate with 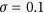 and 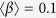 where (c) and (d) represent the cases of 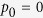 and 0.01, respectively. Comparing Fig. 4(a) with (b) we find that the infected density 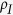 cannot be sustained in (a) but can be sustained in (b), indicating that the threshold for the case of constant *β* is in between 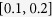, i.e. 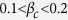. From Fig. 4(c) we see that 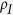 decays to zero much faster than that in Fig. 4(a) with the same 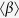. This point can be understood as follows. Because of the fluctuation of 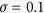, 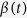 in Fig. 4(c) will be changed around 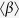. Once it is located in the range 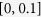, 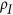 will decay faster than the case of 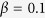 in Fig. 4(a), resulting in the fast decaying observed in Fig. 4(c). However, Fig. 4(d) shows a totally different picture where the recurrent behaviors of non-periodic epidemic patterns can be regenerated by a small but nonzero 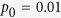, indicating that the time-dependent 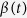 and the nonzero 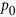 are both the necessary conditions to guarantee the recurrent outbreaks. More detailed dependence on these parameters is shown in Figs 3, 4, 5 in SI.

### Mechanism of non-periodicity in recurrent epidemic patterns

**Figure 5:**
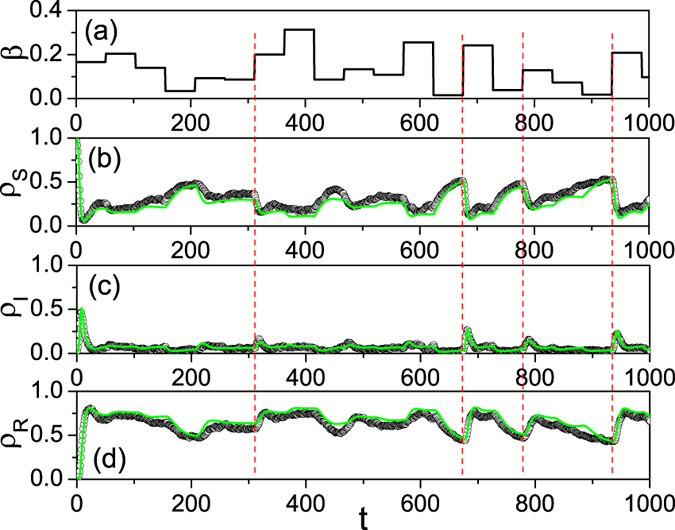
Corresponding relationship between *β*(*t*) and the susceptible, infected, refractory densities with the parameters 〈*β*〉 = 0.1, *σ* = 0.1 and *p*_0_ = 0.01. (**a**) *β* versus *t*; (**b**) 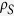 versus *t*; (**c**) 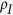 versus *t*; (**d**) 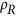 versus *t*. The green solid lines in (**b**–**d**) represent the theoretical results from Eq. (3). The red dashed lines denote some typical positions for epidemic to outbreak.

To reveal the mechanism of the recurrent outbreaks in our network model, we show the corresponding relationship between 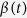 and the susceptible, infected, refractory densities in Fig. 5 where the parameters are taken as 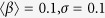 and 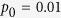. Very interestingly, we find that the largest infection rate *β* does not always induce the epidemic outbreak. Instead, the outbreak in Fig. 5(c) usually occurs at those relatively larger *β*, see the red dashed lines in Fig. 5. From Fig. 5(a,b,d) we notice that all the red dashed lines correspond to those points with both a larger *β* and a larger 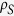 (or a smaller 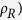. This is reasonable as a larger 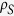 will provide enough population source for the epidemic to grow up and a larger *β* satisfies the condition of 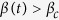. Therefore, a larger infection rate and a larger susceptible density are both the necessary conditions for the recurrent epidemic patterns with non-periodicity. Its theoretical explanation will be given in the section of *Methods*.

### Influence of network structure

**Figure 6:**
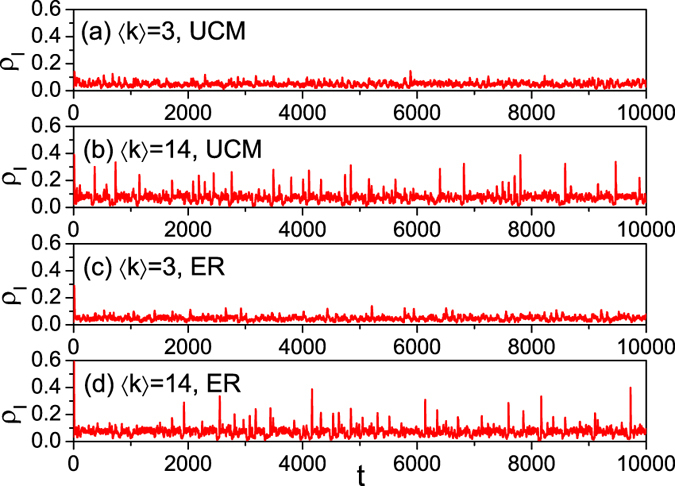
Evolution of *ρ*_*I*_ for different network structures. (**a**,**b**) represent the case of UCM network with 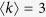 and 14, respectively. (**c**,**d**) represent the case of ER network with 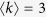 and 14, respectively.

One more key question is how the network topology influences the recurrent outbreaks of epidemic. To answer this question, we first consider the influence of the average degree 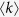. Figure 6(a,b) show the numerical results of two typical cases, i.e. approximately half average degree of Fig. 4 in (a) and approximately double average degree of Fig. 4 in (b). Comparing them with Fig. 4(d), it is easy to see that there is much less outbreaks in Fig. 6(a) but more outbreaks in Fig. 6(b), indicating that larger 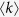 is in favor of the recurrent outbreaks.

Secondly, we consider the influence of degree distribution, i.e. replacing the UCM network with a power-law degree distribution by an Erdös-Rényi (ER) networks with a Poisson distribution^49^. We let the constructed ER network have the same size 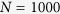 and the same average degree 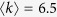 as in Fig. 4. Fig. 6 in SI shows the result. Comparing it with Fig. 4(d), we see that they are similar, except that the recurrent outbreak in UCM network is slightly easier to be observed than in ER network. Furthermore, we have also checked the influence of 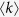 on ER network. Figure 6(c) and (d) show the numerical results, corresponding to Fig. 6(a,b). Comparing Fig. 6(a,b) with (c) and (d), respectively, we see that both (a) and (c) have only a few outbreaks while both (b) and (d) have frequent outbreaks, indicating the robustness to network topologies.

## Discussion

The epidemic spreading on networks is a very hot topic in the field of complex network in recent years, which focuses mainly on the epidemic threshold and how the spreading is influenced by the network structure and other parameters, but to our knowledge, it does not deal with the topic of the recurrent outbreaks of epidemic in complex networks so far. At the same time, the recurrence of influenza has been also paid some attention in a parallel line, which mainly focuses on the mechanism of periodic outbreaks, but it does not deal with the influence of network structures. We here combine these two parallel lines together by presenting a SIRS network model to describe the recurrent epidemic patterns. This work is mainly focused on the non-periodicity observed from the Hong Kong influenza data and other data, in contrast to the previous focus on the periodicity of recurrent epidemic patterns. This understanding to non-periodicity will be useful in a global effort to reduce the impact of a realistic influenza pandemic.

By this network model we reveal that the recurrent outbreaks of epidemic is closely related to three parameters, i.e. the fluctuation of 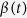, the small infection probability 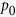 in nature and the average degree 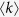 of network. For the first one, a fluctuated 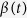 means that it is possible for a smaller 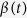 to be followed by a larger 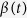. In the time period of the smaller 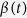, the infected density will be small and thus the susceptible density will be large, which provides enough susceptible source. In the coming period of the larger 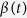, we have 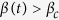 and thus the sufficient susceptible people will guarantee an outbreak. When this condition is satisfied from time to time, we will have the recurrent outbreaks. For the second one, previous studies did not consider 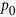 but just initial infected seeds. When the network has an epidemic outbreak, it will come to a decay period until no infected ones in the system, and thus no possibility for a further outbreak. In this sense, a persistent 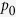 is necessary to ignite another outbreak and is also consistent with practical cases where there is always a small possibility for infected seeds to be generated naturally. For the third one, a smaller average degree means that an infected seed does not have enough neighbors to be infected and thus cannot induce an outbreak. While a larger average degree can provide sufficient neighbors to be infected and thus can induce an outbreak. In sum, these three parameters work together to generate the recurrent outbreaks of epidemic. After understanding this point, we expect that an effective way to control the recurrent outbreaks can be also found by considering these three factors. For example, we can reduce 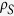 to a small quantity by increasing 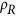 as large as possible by vaccinating more and more people.

## Methods

### Epidemic spreading on the network model of recurrent epidemic patterns

We take the uncorrelated configuration model (UCM) as an example. We first construct the UCM network with a power-law degree distribution 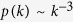 by following ref. 48. We let its size be 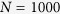, average degree be 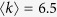, and let *k* be limited in the range 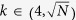. Then, we consider the case that each node of the UCM network is occupied by a person and take the SIRS model for the epidemic spreading, see the schematic plot in Fig. 3. We let a susceptible person have two ways to be infected. One is the infection by a small probability 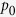, representing the natural infection from environment or unknown reasons. The other is the infection by a contagious rate *β*, representing the infection from contacting with an infected person. When a susceptible node has 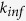 infected neighbors, it will become infected with probability 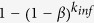. At the same time, the infected node will decay into a refractory one with probability *μ*. For the process from refractory to susceptible state, ref. 47 assumed that a person will stay at the refractory state for a constant time *τ* and then go back to the susceptible state. However, in reality, individuals may have different habitus and thus may need different *τ* to recover. To overcome this defect, instead of the fixed *τ*, we here let each refractory person have a small probability *δ* to recover from the refractory to susceptible state. In numerical simulations, we fix 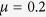 and 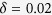.

We choose the dependence of 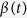 on time by the following way: we divide the time *t* into *T* intervals with equal length and let *T* = 52, corresponding to the 52 weeks in one year. We let 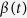 be a constant in each interval and different constants in different intervals. The value of the constant is randomly chosen from the Gaussian distribution with average 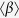 and standard deviation *σ*. Once a negative 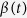 is chosen, we discard it and then choose a new one.

### A theoretical analysis based on Markov dynamics

Let 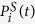, 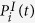, and 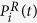 be the probability for person *i* to be in the state of *S*, *I* and *R* at time *t*, respectively. Then we have 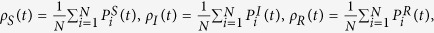 where 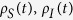 and 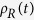 represent the densities of susceptible, infected, and refractory agents at time *t*, respectively. Let 
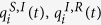
 and 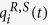 be the transition probability from the state *S* to *I*, *I* to *R* and *R* to *S*, respectively. By the Markov chain approach^5^,^50^ we have


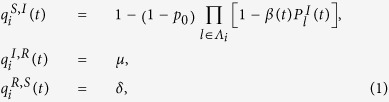


where 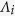 represents the neighbors of node *i*. The term 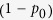 in Eq. (1) represents the probability that node *i* is not infected by the environment. While the term 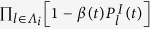 is the probability that node *i* is not infected by the infected neighbors. Thus, 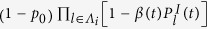 is the probability for node *i* to be in susceptible state. Based on this analysis, we formulate the following difference equation model to help gain insights into the network model’s dynamics


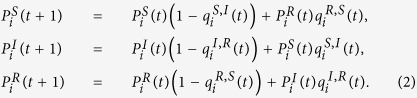


The first term on the right-hand side of the first equation of Eq. (2) is the probability that node *i* is remained as susceptible state. The second term stands for the probability that node *i* is changed from the refractory to susceptible state. Similarly, we have the same explanation for the second and third equations of Eq. (2). Substituting Eq. (1) into Eq. (2) we obtain


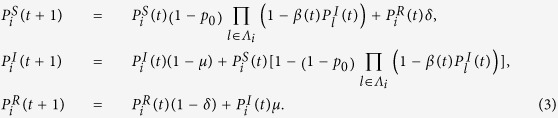


Instead of getting the analytic solution of Eq. (3), we solve Eq. (3) by numerical integration. To conveniently compare with the model in section *Results*, we use the same set of time-dependent 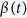 to both the model and Eq. (3). In this way, we can obtain the corresponding theoretical results. The green solid lines in Fig. 5(b–d) show the theoretical results from Eq. (3). Comparing them with the experimental results “circles” there, we see that they are consistent with each other very well, indicating that Eq. (3) can completely explain the numerical results.

## Additional Information

**How to cite this article**: Zheng, M. *et al.* Non-periodic outbreaks of recurrent epidemics and its network modelling. *Sci. Rep.* **5**, 16010; doi: 10.1038/srep16010 (2015).

## Supplementary Material

Supplementary Information
