# Supplementary material for: Non-periodic outbreaks of recurrent epidemics and its network modelling
Source: Sci Rep. 2015 Nov 2;5:16010. doi: 10.1038/srep16010 (PMC4629194; doi:10.1038/srep16010)
Supplement: Supplementary Information [file srep16010-s1.pdf]

# Non-periodic recurrent outbreaks of epidemic and its network modelling

Muhua Zheng, Chaoqing Wang, Jie Zhou, Ming Zhao, Shuguang Guan, Yong Zou, and Zonghua Liu

## I. SUPPLEMENTARY TABLES

TABLE I: Detection of influenza viruses in respiratory specimens from 2010 to 2013, provided by the Centre for Health Protection, Department of Health, the Government of the Hong Kong Special Administrative Region.

| year | No. of specimen tested | Type A: H3N2 | Type A: H1N1 | Type A: H5N1 | Type B | Type C |
|---|---|---|---|---|---|---|
| 2010 | 71130 | 4261 | 2650 | 2 | 2227 | 0 |
| 2011 | 61103 | 1290 | 3779 | 0 | 1299 | 1 |
| 2012 | 80391 | 9043 | 202 | 4 | 3590 | 1 |
| 2013 | 85014 | 3622 | 2709 | 0 | 564 | 0 |

## II. SUPPLEMENTARY FIGURES

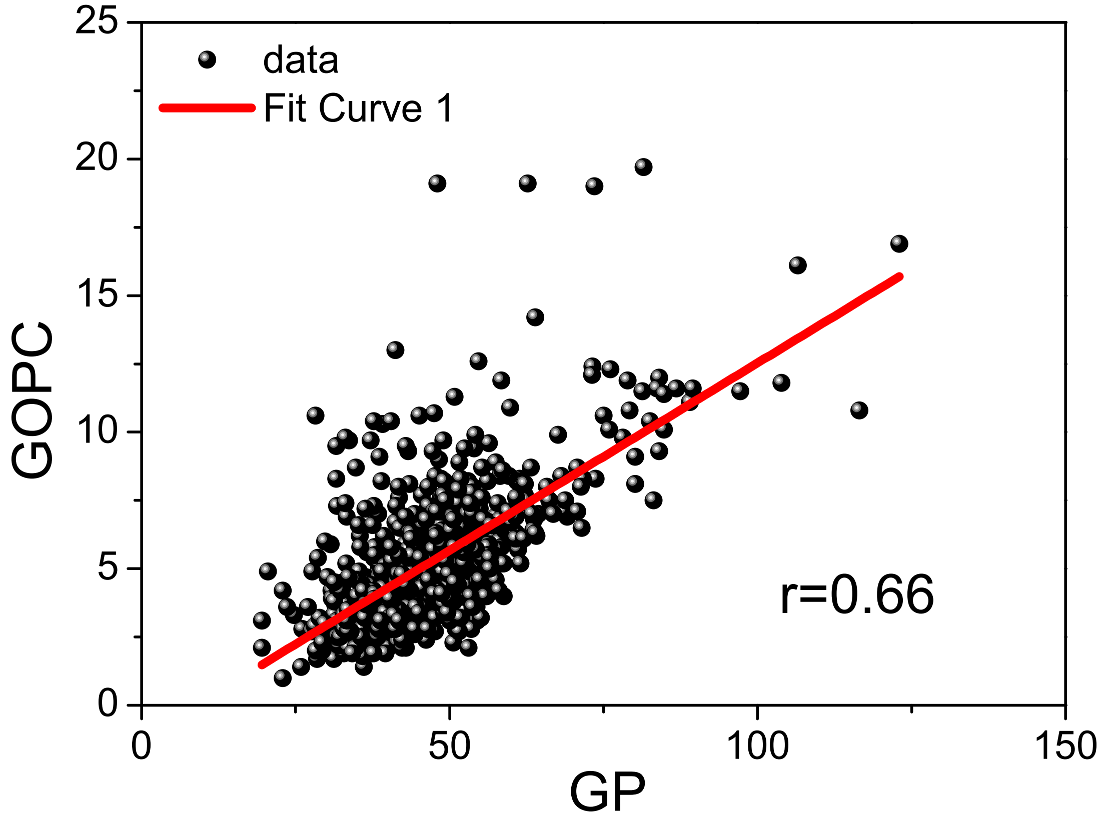


FIG. 1: (color online.) **Correlation between GOPC and GP**. We let the variable $C$ in Fig. 1(a) of main text be $x_i$ and the variable $C$ in Fig. 1(b) of main text be $y_i$. Then we can define the correlation coefficient as $r = \frac{\sum_{i=1}^{n}(x_i-\overline{x})(y_i-\overline{y})}{\sqrt{\sum_{i=1}^{n}(x_i-\overline{x})^2 \cdot \sum_{i=1}^{n}(y_i-\overline{y})^2}}$, where the $\overline{x}$ and $\overline{y}$ represent the average of $x_i$ and $y_i$, respectively. We find that $r \approx 0.66$, indicating a strong correlation between the time series of GOPC and GP.

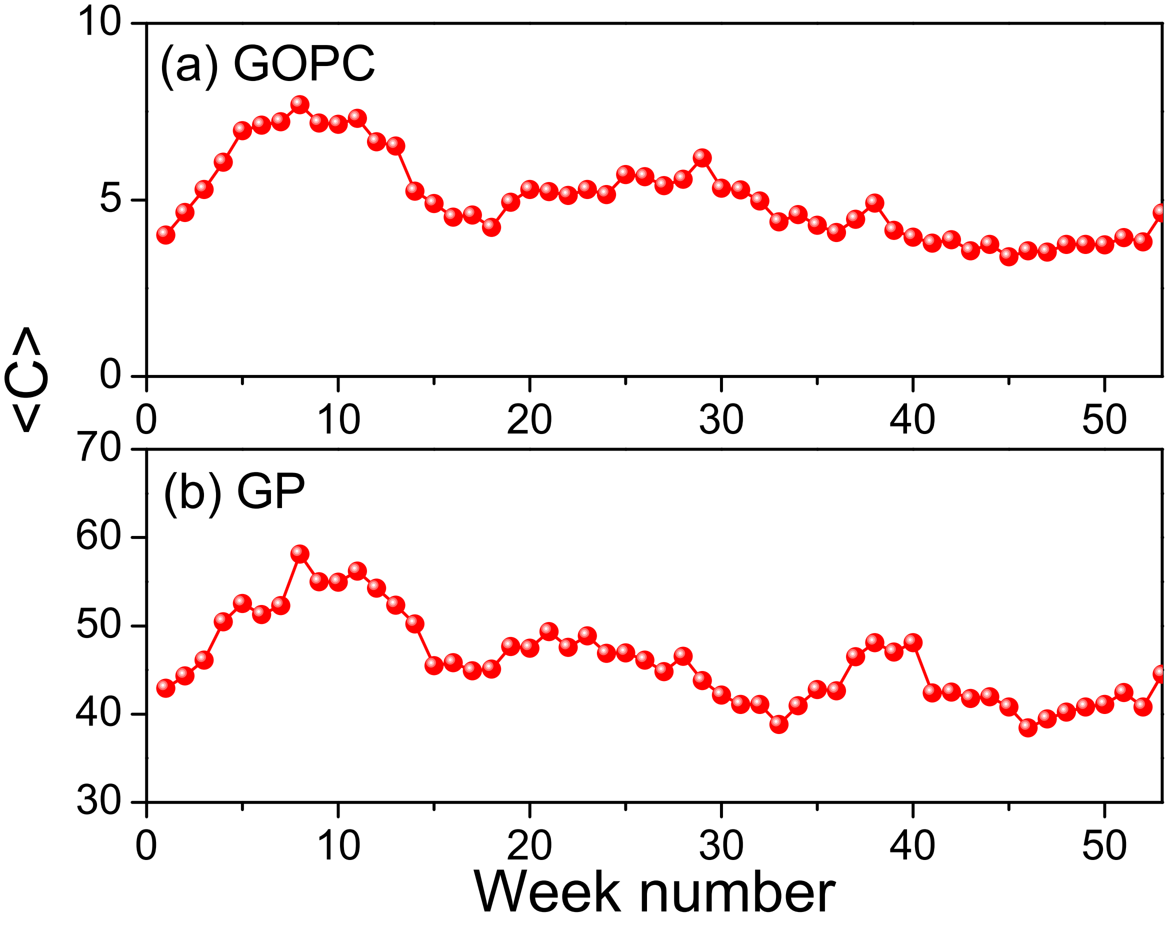


FIG. 2: (color online.) **Average weekly consultation rates of influenza-like illness (per 1000 consultations) over the 16 years in the cases of (a) and (b) of Fig. 1 in the main text**. (a) and (b) represent the cases of the General Out-Patient Clinics (GOPC) and the General Practitioners (GP), respectively, which correspond to the cases of (a) and (b) of Fig. 1 in the main text. We see that the largest $\langle C \rangle$ in both (a) and (b) appears at the eighth week, indicating that most of the outbreaks happens in winter or spring. We also notice that $\langle C \rangle$ is oscillatory but not an unimodal distribution, supporting the feature of non-periodicity again.

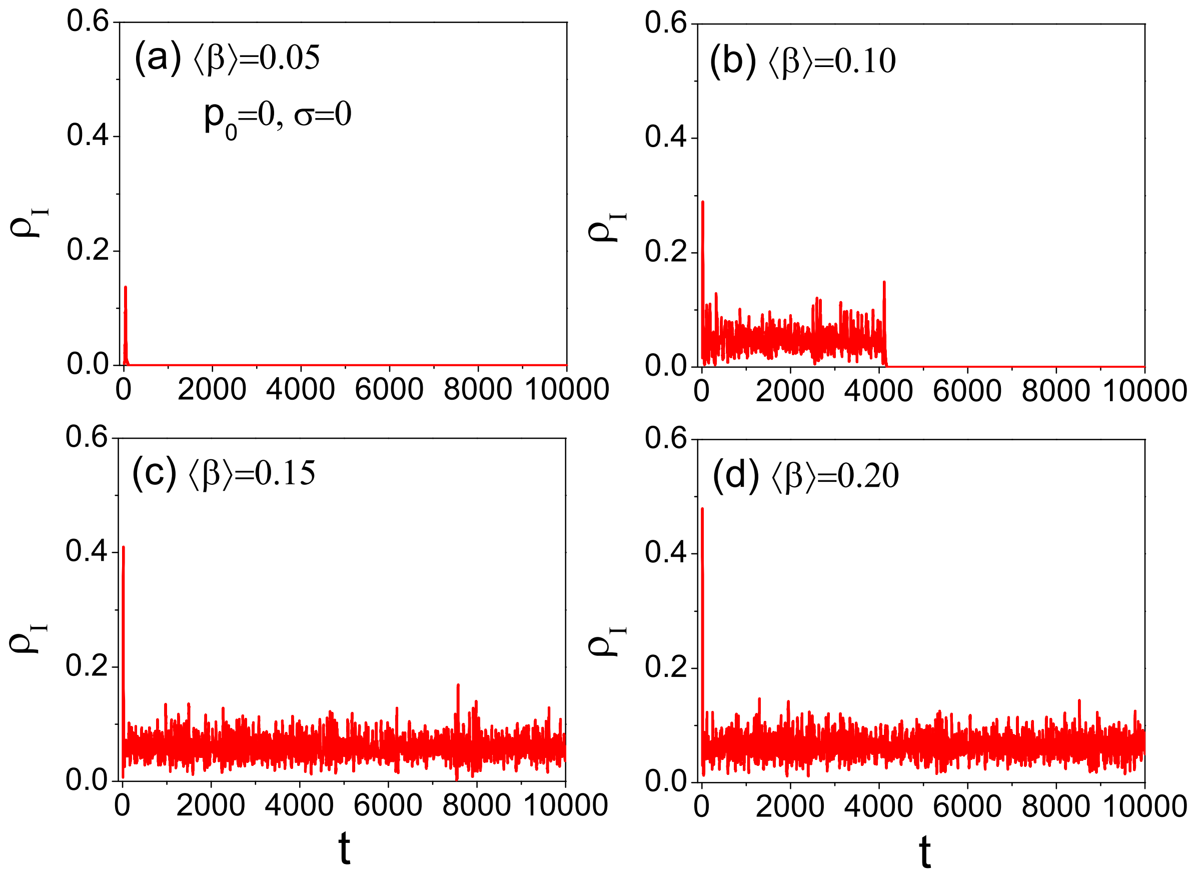


FIG. 3: (color online.) **Evolution of infected density $\rho_I$ for the case of constant infection rate with $p_0 = 0$ and $\sigma = 0$.** (a) Case of $\beta = 0.05$; (b) Case of $\beta = 0.1$; (c) Case of $\beta = 0.15$; (d) Case of $\beta = 0.2$. We see that the epidemic cannot be sustained in the cases of (a) and (b) but can be sustained in the cases of (c) and (d), indicating that the threshold $\beta_c$ is in between $[0.1, 0.15]$.

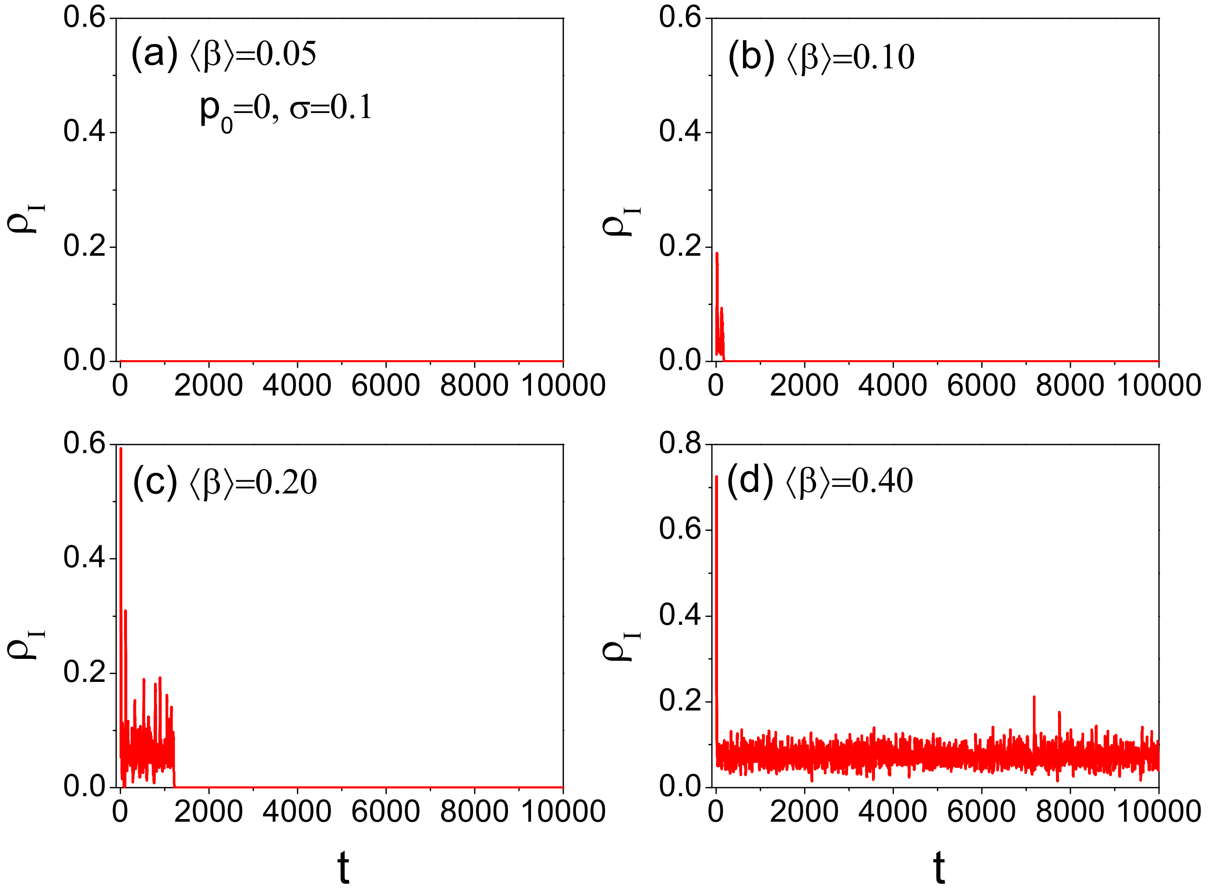


FIG. 4: (color online.) **Evolution of infected density $\rho_I$ for the case of time-dependent infection rate with $\sigma = 0.1$ but no $p_0$.** (a) Case of $\langle\beta\rangle = 0.05$; (b) Case of $\langle\beta\rangle = 0.1$; (c) Case of $\langle\beta\rangle = 0.2$; (d) Case of $\langle\beta\rangle = 0.4$. We see that the epidemic cannot be sustained in the cases of (a)-(c) but can be sustained in the case of (d). From Fig. 3 we know that the threshold $\beta_c$ is less than $0.15$. We here notice that $\langle\beta\rangle = 0.2$ in (d) is greater than $\beta_c$, but its $\rho_I$ cannot be sustained. Why? The reason is that the fluctuation $\sigma = 0.1$ will make $\beta(t)$ be sometimes less than $\beta_c$. Once it happens, $\rho_I$ will be decreased to zero and stop there forever.

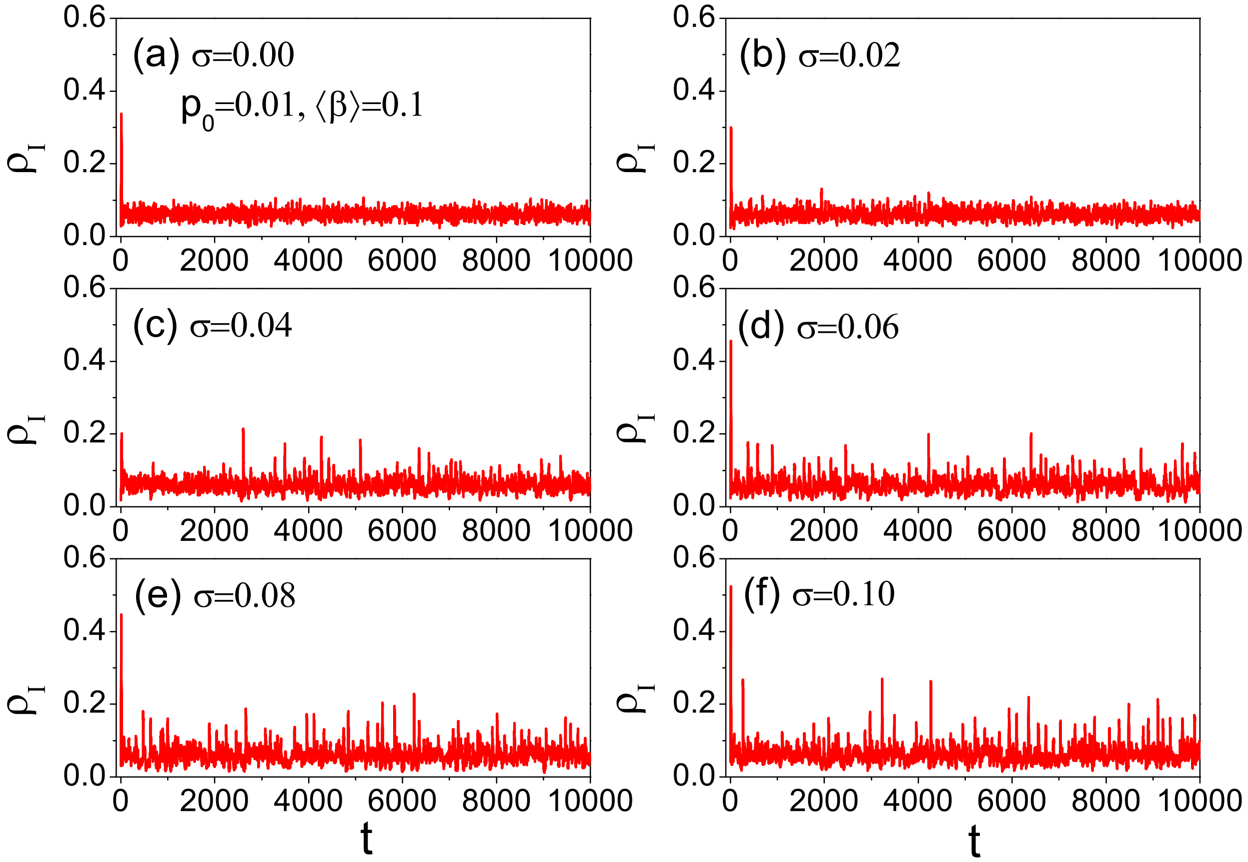


FIG. 5: (color online.) **Influence of the fluctuation $\sigma$ on the outbreaks for the case of both time-dependent infectious rate $\langle\beta\rangle = 0.1$ and nonzero $p_0 = 0.01$.** (a) Case of $\sigma = 0.0$; (b) Case of $\sigma = 0.02$; (c) Case of $\sigma = 0.04$; (d) Case of $\sigma = 0.06$; (e) Case of $\sigma = 0.08$; (f) Case of $\sigma = 0.1$. We see that the epidemic outbreaks cannot be sustained in the cases of (a) and (b) for too small fluctuation $\sigma$ but can be sustained in the case of (c) to (f) for larger $\sigma$, indicating that there is a threshold $\sigma_c$ for epidemic to outbreak recurrently. Comparing with Fig. 3 and Fig. 4 we find that the value of $\langle\beta\rangle$ needed for recurrent outbreaks is much smaller in Fig. 5 than that in Figs. 3 and 4, indicating that both the time-dependent infectious rate $\beta(t)$ and nonzero $p_0$ are accounted for the recurrent outbreaks.

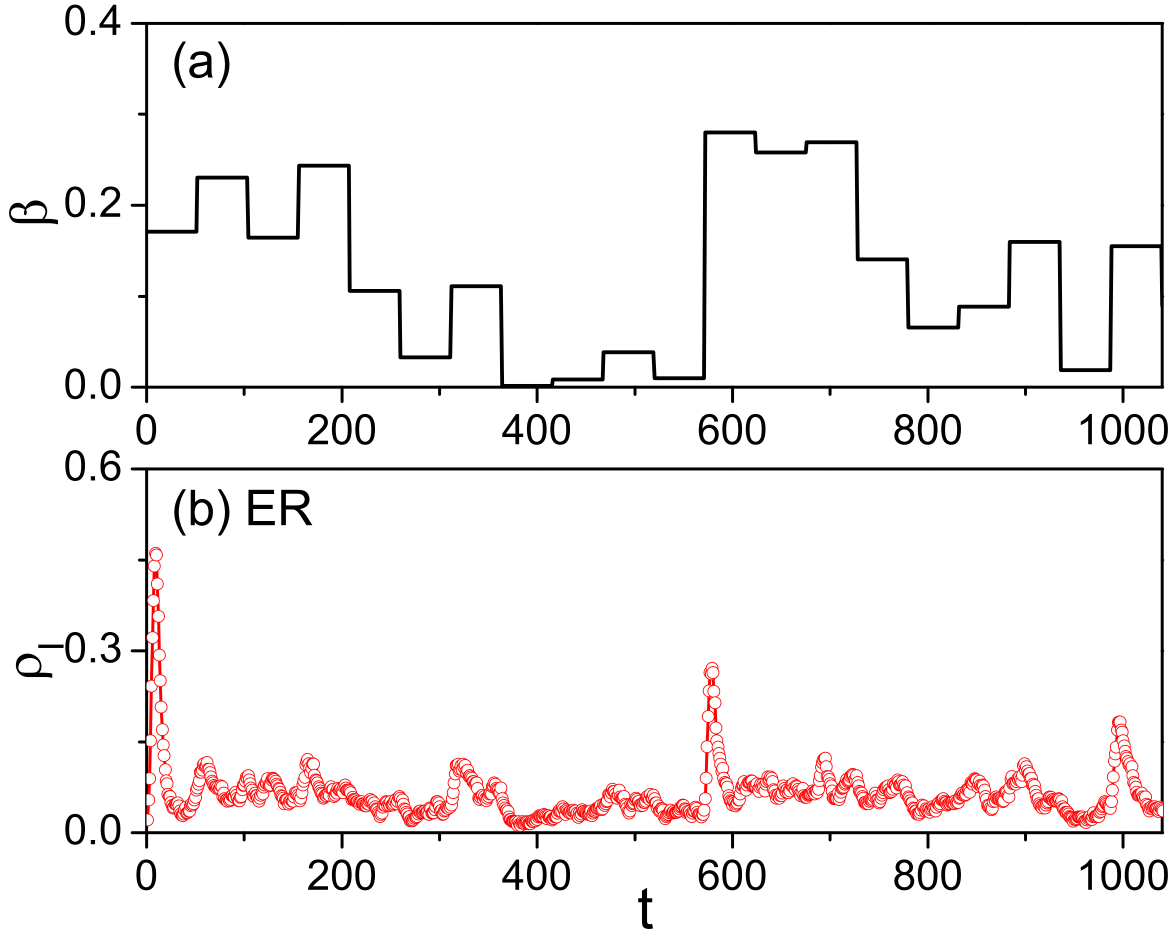


FIG. 6: (color online.) **Recurrent outbreaks in an ER network with** $p_0 = 0.01$. This ER network has the same size and average degree with the UCM network in main text, i.e. $N = 1000$ and $\langle k \rangle = 6.5$. (a) $\beta$ versus $t$ with $\langle \beta \rangle = 0.1$ and $\sigma = 0.1$. (b) $\rho_I$ versus $t$. We see that there is also recurrent outbreaks in (b), indicating the robustness of our model to network topologies.
